# Magnetoactive Acoustic Topological Transistors

**DOI:** 10.1002/advs.202201204

**Published:** 2022-04-25

**Authors:** Kyung Hoon Lee, Hasan Al Ba'ba'a, Kunhao Yu, Ketian Li, Yanchu Zhang, Haixu Du, Sami F. Masri, Qiming Wang

**Affiliations:** ^1^ Sonny Astani Department of Civil and Environmental Engineering University of Southern California Los Angeles CA 90089 USA

**Keywords:** acoustic metamaterials, field‐effect transistor, quantum valley hall effect, topological acoustics

## Abstract

Topological field‐effect transistor is a revolutionary concept that physical fields are used to switch on and off quantum topological states of the condensed matter. Although this emerging concept has been explored in electronics, how to realize it in the acoustic realm remains elusive. In this work, a class of magnetoactive acoustic topological transistors capable of on‐demand switching on and off topological states and reconfiguring topological edges with external magnetic fields is presented. The key mechanism is to harness magnetic fields to tune air‐cavity volumes within acoustic chambers, thus breaking or preserving the inversion symmetry to manifest or conceal the quantum valley Hall effect. To switch the topological transport beyond the in‐plane routes, a magneto‐tuned non‐topological band gap to allow or forbid the wave transport out‐of‐plane is harnessed. With the reversible magnetic control, on‐demand switching of topological routes to realize topological field‐effect waveguides and wave regulators is demonstrated. Analogous to the impact of semiconductor transistors on modern electronics, this work may expand the scope of topological acoustics by achieving unprecedented functions in acoustic modulation.

## Graphical abstract

Although topological field‐effect transistor has been explored in electronics, how to realize it in acoustics remains elusive. This study presents a class of magnetoactive acoustic topological transistors capable of on‐demand switching on and off topological states with external magnetic fields. Integrating magneto‐tunable topological and non‐topological band gaps, in‐plane and out‐of‐plane conductive routes can be on‐demand reconfigured without rebuilding structures.

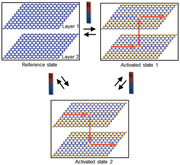

## 1 Introduction

Field‐effect transistors (FETs), a class of devices that use gate voltages to control electric current flows, were invented around 90 years ago^[^ ^1^ ^]^ and eventually inspired the innovation of semiconductor transistors for the Nobel Prize in Physics in 1956.^[^ ^2^ ^]^ FETs have become one of the most important modern electronic devices for broad applications such as switches, modulators, amplifiers, and stabilizers.^[^ ^3^ ^]^ Recently, FETs have witnessed a revolution because of the innovation of topological field‐effect transistors (TFETs)^[^ ^4^, ^5^, ^6^, ^7^, ^8^ ^]^ by integrating quantum topological effects, such as the quantum Hall effect^[^ ^9^, ^10^ ^]^ and quantum spin Hall effect,^[^ ^11^, ^12^ ^]^ into FETs. In these TFETs, physical fields are utilized to on‐demand switch on and off the quantum topological effects and the corresponding topological edges that serve as switchable conductive paths for low‐dissipation flows of charges and spins, thus, to achieve low‐energy logic circuits.^[^ ^4^, ^5^, ^6^, ^7^, ^8^ ^]^ Although the concept of TFET has been explored in the field of electronics,^[^ ^4^, ^5^, ^6^, ^7^, ^8^ ^]^ how to realize it in the field of acoustics remains elusive. Analogous to the impact of semiconductor transistors on modern electronics,^[^ ^2^ ^]^ acoustic topological field‐effect transistors, if successfully invented, are expected to drastically expand the application scope of topological acoustics^[^ ^13^, ^14^, ^15^ ^]^ by achieving unprecedented functions in acoustic modulation.

**Figure 1:**
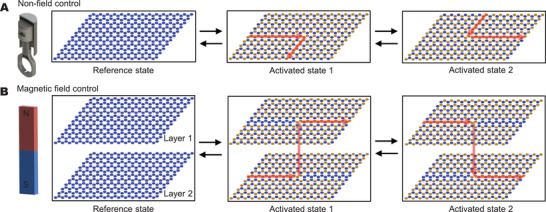
Comparison between A) the existing active acoustic topological insulators and B) the proposed magnetoactive acoustic topological transistors (MATTs). Two innovations of the proposed MATTs: (1) employing a physical field (i.e., magnetic field) to achieve on‐demand control, and (2) realizing on‐demand switching of both in‐plane and out‐of‐plane routes.

Inspired by the topological insulators in condensed matter physics,^[^ ^16^, ^17^, ^18^ ^]^ the studies of topological acoustics have been exploring analogous quantum topological states in the realm of acoustics.^[^ ^13^, ^14^, ^15^ ^]^ The topological acoustics have been realized using a number of strategies, such as circulating fluids or gauge flux to break the time‐reversal symmetry,^[^ ^19^, ^20^ ^]^ harnessing strong coupling ring resonators as an analog to Floquet insulators,^[^ ^21^, ^22^ ^]^ utilizing valley‐Hall or pseudospin effects in 2D^[^ ^21^, ^23^, ^24^, ^25^, ^26^ ^]^ and 3D sonic crystals,^[^ ^27^, ^28^, ^29^ ^]^ identifying Weyl points in phononic crystals,^[^ ^30^, ^31^, ^32^ ^]^ and constructing sonic crystals with higher‐order topological states.^[^ ^33^, ^34^, ^35^, ^36^, ^37^, ^38^, ^39^, ^40^ ^]^ In these existing topological acoustics metamaterials, delicate periodic architectures are required to be judiciously designed and precisely fabricated; thus, turning on and off the acoustic topological states and reconfiguring the topological routes (**Figure** **1**A), without rebuilding the structures, are typically challenging.^[^ ^41^, ^42^, ^43^ ^]^ Although physical fields (such as electric and magnetic fields) have been employed to realize appealing active non‐topological acoustic metamaterials capable of non‐local, rapid, and reversible modulation of acoustic properties,^[^ ^44^, ^45^, ^46^, ^47^, ^48^, ^49^, ^50^ ^]^ how to utilize physical fields to switch topological acoustics remains elusive (Figure 1B). Besides, due to high complexity in architectures,^[^ ^27^, ^28^, ^35^, ^36^, ^37^ ^]^ existing active acoustic topological insulators only deal with in‐plane routes (Figure 1A); on‐demand switching of topological acoustic transport beyond the conventional in‐plane routes to out‐of‐plane routes is still a big challenge in the field (Figure 1B).

Here, we report a class of magnetoactive acoustic topological transistors (MATTs) capable of on‐demand switching on and off topological states, and reconfiguring acoustic conductive routes among orthogonal directions, both in‐plane and out‐of‐plane, with external magnetic fields (Figure 1B). The proposed structure consists of air‐cavity chambers connected in a hexagonally periodic architecture. The key mechanism in realizing MATTs is to use magnetic fields to tune the air‐cavity volume differently in each chamber, thus breaking or preserving the inversion symmetry to manifest or conceal the quantum valley Hall effect.^[^ ^13^, ^14^, ^15^ ^]^ To switch the topological transport beyond the in‐plane routes, we harness a magneto‐tuned non‐topological band gap to allow or forbid the wave transport out‐of‐plane. The magnetic control is rapid and reversible, thus enabling on‐demand switching of topological conductive routes that feature high immunity to structural defects. Integrating the orthogonal controls (in‐plane and out‐of‐plane), we further exploit the MATTs to realize topological field‐effect waveguides and wave regulators.

## 2 Results

### 2.1 Concept of Magnetoactive Acoustic Topological Transistors (MATTs)

**Figure 2:**
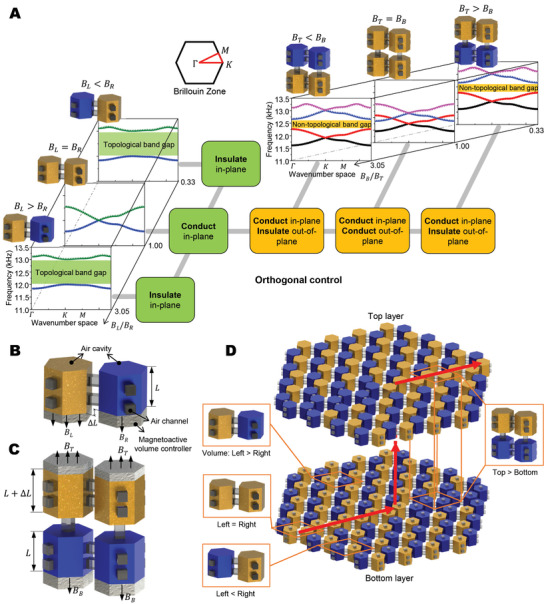
Concept of the magnetoactive acoustic topological transistor (MATT). A) Concept of orthogonal control using the magneto‐tunable dispersion relationships of two‐cavity units and four‐cavity units. Opening and closing of topological band gaps (green shaded regions) in two‐cavity units control the insulation and conduction of in‐plane wave routes. Opening and closing of the non‐topological band gaps (orange shaded regions) in four‐cavity units control the insulation and conduction of out‐of‐plane wave routes. B) Schematic of a four‐cavity unit with magneto‐active volume controllers. The magnetic fields applied to the top and bottom volume controllers are denoted as *B_T_* and *B_B_*, respectively. C) Schematic of a two‐cavity unit with magneto‐active volume controllers (in gray). The magnetic fields applied to left and right volume controllers are denoted as *B_L_* and *B_R_*, respectively. D) The proposed conceptual model of MATT with two‐layer hexagonal‐prism‐shaped air‐cavity networks. The insets illustrate three types of two‐cavity units and a four‐cavity unit.

We plan to study the topological acoustics within chambers where the acoustic wave transports within connected air‐cavity networks. By harnessing magneto‐actuated modulation of volumes of the air‐cavity elements, we will realize the topological transport of the acoustic waves. To demonstrate the concept of MATTs, we start with the introduction of the dispersion relationships of unit‐cell elements for MATT devices (**Figure** **2**A). We first consider two prism air‐cavities laterally connected to form a two‐cavity unit (Figure 2A left part and Figure 2B). Each air cavity chamber houses a magneto‐active volume controller whose height can be adjusted by a magnetic field, thus regulating the air‐cavity volume (Figures 2B). The applied magnetic fields on the left and right cavities of the two‐cavity unit are denoted as *B_L_* and *B_R_*, respectively. The air‐cavity volume can be enlarged by applying a higher magnitude of the magnetic field. Depending on the relative values of *B_L_* and *B_R_*, two‐cavity units can be classified into three types whose band structures are shown in the left part of Figure 2A: When *B_L_* > *B_R_* or *B_L_* < *B_R_*, a topological band gap (i.e., green shaded region in Figure 2A) at the Dirac point (K‐point in the Brillouin zone) is opened, indicating that the wave is forbidden within the topological band gap. When *B_L_* = *B_R_*, the topological band gap is closed at the Dirac point, permitting the wave to conduct within the topological band gap.

Next, we pick two two‐cavity units vertically connected to form a four‐cavity unit (Figure 2A right part and Figure 2C). We focus on the laterally conductive phase (*B_L_* = *B_R_*) to reveal the vertical interactions between two two‐cavity units. The Magneto‐active volume regulators are installed within both the top and bottom air cavities (Figure 2C). Since we only consider the air cavities along with the laterally conductive phase, the laterally connected air‐cavities have the same air volume (i.e., *B_L_* = *B_R_*). We denote the applied magnetic fields on the top and bottom cavities as *B_T_* and *B_B_*, respectively. Depending on the relative values of *B_T_* and *B_B_*, we can classify four‐cavity units into three types whose band structures are shown in the right part of Figure 2A. When *B_T_* > *B_B_* or *B_T_* < *B_B_*, a non‐topological band gap (i.e., orange shaded region) is opened to prevent the out‐of‐plane wave motion; while when *B_T_* = *B_B_*, the non‐topological band gap is closed to allow the wave to move out of the plane.

By integrating the two‐cavity units and four‐cavity units displayed in Figure 2A, we propose a conceptual model for MATTs as follows: It consists of two layers of hexagonally connected prism air‐cavities, where air‐cavities in the bottom layer are linked to the vertical neighbors in the top layer by air channels (Figure 2D). On one hand, in the bottom or top layer, the breaking of the inversion symmetry to manifest the quantum valley Hall effect^[^ ^13^, ^14^ ^]^ can be achieved by applying designated magnetic fields to different cavities. As shown in the bottom layer of Figure 2D, the central orange path is a topologically conductive edge (*B_L_*/*B_R_* = 1) that is surrounded by topologically insulative phases (*B_L_*/*B_R_* > 1 and *B_L_*/*B_R_* < 1). The topological edge path can be turned on and off by modulating the magnetic field ratio *B_L_*/*B_R_* in each two‐cavity unit within the air‐cavity network, thus mimicking the electronic TFETs.^[^ ^4^, ^5^, ^6^, ^7^, ^8^ ^]^ In the “on” state, the acoustic flow conducts through the topological edge path, while modulating the magnetic fields would turn the system “off” as there would be no topological edges. On the other hand, along the in‐plane conductive path, the conduction or insulation between vertical air‐cavity elements can be controlled by another magnetic field ratio *B_T_*/*B_B_*: allowing out‐of‐plane conduction when *B_T_*/*B_B_* = 1 and forbidding out‐of‐plane conduction when *B_T_*/*B_B_* ≠ 1 (Figure 2A,D). Overall, the structure can be orthogonally controlled by two magnetic field ratios: *B_L_*/*B_R_* and *B_T_*/*B_B_*. The effective frequency range is determined by the overlap of the topological band gap (i.e., green shaded region) and the non‐topological band gap (i.e., orange shaded region) (Figure 2A).

### 2.2 Magnetoactive Switching of In‐Plane Conductive Routes

**Figure 3:**
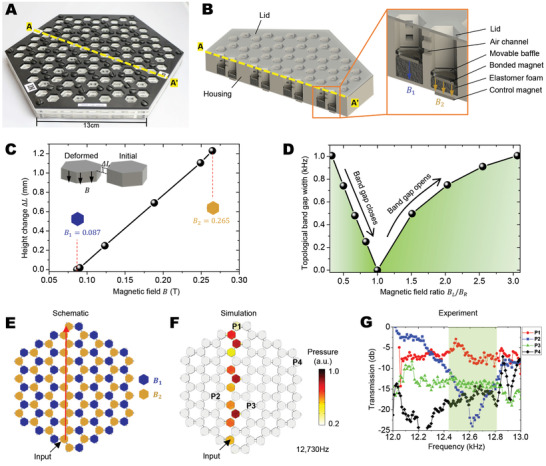
Experimental realization of magnetoactive switching of in‐plane wave routes. A) A single‐layer air‐cavity chamber structure constructed with patterned acrylic sheets. B) Computer‐aided design (CAD) models to show the cutaway view of the air‐cavity chamber. The zoom‐in inset shows the cutaway view of a two‐cavity unit. C) The experimentally measured height change Δ*L* of the volume controller as a function of the applied magnetic field. The inset shows the deformation of the volume controller. Magnetic fields *B*_1_ = 0.087 and *B*_1_ = 0.265 are corresponding to Δ*L* ≈ 0 and Δ*L* = 1.21 mm, respectively. D) The numerically calculated topological band gap width in a function of the magnetic field ratio *B_L_*/*B_R_*. E) Top‐view schematics of air‐cavity patterns. Blue and orange cavities are corresponding to applied magnetic fields *B*_1_ = 0.087*T* and *B*_2_ = 0.265*T*, respectively. F) Numerical simulations of the acoustic pressure within the air‐cavity network. G) Experimentally measured acoustic transmission at cavities P1, P2, P3, and P4 in functions of frequencies. Light green shaded regions (12.4‐12.82 kHz) indicate the effective frequency region.

We first experimentally realize the in‐plane MATTs by constructing an air‐cavity chamber with layers of patterned acrylic sheets (**Figure** **3**A,B and S1, Movie S1, Supporting Information). Hexagonal‐prism‐shaped air‐cavity chambers are connected by air channels to form a hexagonal network. In each air‐cavity chamber, a magnet‐bonded elastomer foam is installed as the volume controller, whose height can be reversibly tuned by an externally controlled magnetic field (Figure 2B). The elastomer foam can be reversibly actuated to deform by more than 2 mm for more than 200 cycles (Figure S1C, Supporting Information). Under the background magnetic field *B*_1_ = 0.087*T*, the elastomer foam is almost undeformed. When a magnetic field of *B*_2_ = 0.265*T* is applied to a selected cavity via a control magnet, the elastomer foam is deformed by a height of 1.21 mm, and the cavity volume is enlarged (Figure 3C). The topological band gap width can be on‐demand tuned from 0 to 1 kHz by modulating the magnetic field ratio *B_L_*/*B_R_* from 1 (i.e., *B_L_* = *B_R_* = *B*_2_) to 0.33 (i.e., *B_L_* = *B*_1_ and *B_R_* = *B*_2_), or from 1 (i.e., *B_L_* = *B_R_* = *B*_2_) to 3.05 (i.e., *B_L_* = *B*_2_ and *B_R_* = *B*_1_) (Figures 3D and S2, Supporting Information). With programmed magnetic fields designated to different air‐cavity chambers, a topological edge path will be on‐demand exhibited to allow the topological conduction of acoustic waves (edge modes illustrated in Figure S3, Supporting Information). As an example shown in Figure 3E, an I‐pattern topological path is prescribed by designating the control magnets under the orange air‐cavity. The prescribed topological path is verified by a numerical simulation that shows much higher acoustic pressure along the topological path, compared to other regions (12.73 kHz in Figure 3F, other frequencies in Figure S4A,B, Supporting Information). To further verify the topological path, we conduct experiments by inputting the acoustic signals from the same air cavity and measuring the acoustic transmission at multiple cavities either on or away from the topological path (P1–P4, Figures 3G and S6, Supporting Information). Based on the band gap analyses in Figure 2A, the expected effective frequency range should be within the topological band gap (12‐13 kHz); thus, we carry out experiments within 12–13 kHz. The experimental results show that within a certain frequency range the acoustic transmission is high if the measured cavity is on the path (P1), and low elsewhere (P2–P4) (Figure 3G). In Figure 3G, the effective frequency range is 12.4–12.82 kHz, which is narrower than the topological band gap. It is primarily because the number of the air‐cavities in the experimental setting is relatively small; a structure with sufficient air‐cavities (so‐called “infinitely periodic”) would widen the frequency range to almost match the topological band gap. Note that removing the magnetic actuation would result in turning off the topological edge path (i.e., all blue cavities). Turning on and off the topological edge path can be realized by simply modulating the applied magnetic fields, without rebuilding the structures.

The concept of MATT can be harnessed to realize reversible switching of in‐plane topological waveguides. Figure S6, Supporting Information, illustrates switching among three topological waveguides (I, V, and Z patterns) by reconfiguring the air‐cavities with modulated magnetic actuation (Movie S2, Supporting Information). Both simulations and experiments show that these three topological waveguides are effective through 12.6–12.82 kHz (Figures S4 and S6, Supporting Information). Notably, topological acoustic transport is robust and immune to geometrical defects.^[^ ^13^, ^14^, ^26^ ^]^ Experiments show that the acoustic conduction along the topological edge path is still effective when two air cavities on the Z‐pattern path are deactivated (Figure S7, Supporting Information in comparison to Figure S6G–I, Supporting Information).

### 2.3 Magnetoactive Switching of Out‐of‐Plane Conductive Routes

**Figure 4:**
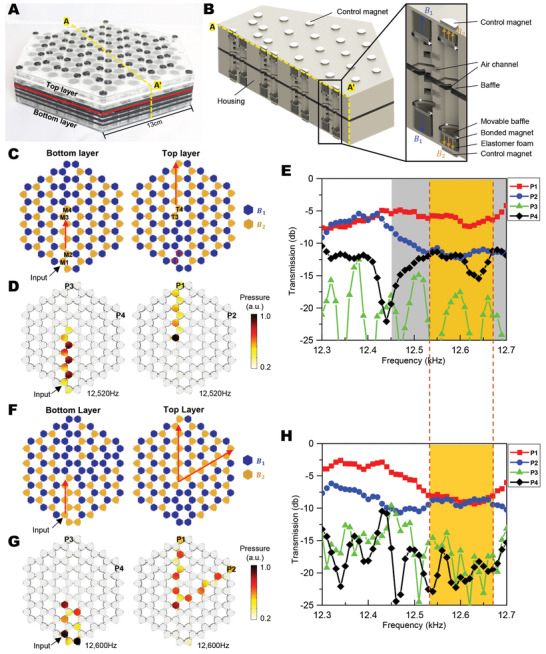
Experimental realization of magnetoactive switching of out‐of‐plane wave routes. A) A double‐layer air‐cavity chamber structure constructed with patterned acrylic sheets. B) A CAD model to show the cutaway view of the air‐cavity chamber. The zoom‐in inset shows the cutaway view of a four‐cavity unit. C–H) Realization of (C‐E) I pattern and (F‐H) Y pattern out‐of‐plane topological waveguides. (C and F) Schematics of the bottom and top layers. (D and G) numerical simulations of the acoustic pressure. (E and H) Experimentally measured acoustic transmission of cavities P1, P2, P3, and P4 in functions of frequencies.

We further integrate the topological band gap and the non‐topological band gap to on‐demand switch the wave routes both in‐plane and out‐of‐plane (Figure 2A,D). To experimentally realize the concept, we stack two single‐layer structures via air‐channel connectors (**Figures** **4**A,B and S8, Supporting Information). The magnetoactive volume controllers are located on the top and bottom in the top and bottom air cavities, respectively (Figure 4B). Depending on the relative values of *B_T_* and *B_B_* (recall Figures 2A and Figure S9, Supporting Information), we can on‐demand allow or forbid the wave transport out‐of‐plane. Here, we present reversible switching between two out‐of‐plane topological waveguides (I and Y patterns, Figure 4C–H, Movie S3, Supporting Information). Taking the I‐pattern waveguide as the starting example, we employ magnetic fields *B*_1_ and *B*_2_ to activate the cavities into a pattern shown in Figure 4C. If an acoustic signal within the non‐topological band gap (12.27–12.65 kHz in Figure 4C) is input in cavity M1 on the bottom layer, the wave signal is expected to travel along the orange path on the bottom layer and then move out of the plane to the orange path on the top layer (following red arrow in Figure 4C). The reason is as follows: Along the orange path on the bottom layer, air cavities M1 and M2 form a two‐cavity unit of the same volume; thus, the wave signal can travel from M1 to M2 (Figure 2A). Cavities M1, M2, T1, and T2 form a four‐cavity unit whose band gap is opened within 12.27–12.65 kHz; thus, the wave signal cannot move from cavities M1 and M2 to cavities T1 and T2 (Figure 2A). When the wave signal travels to cavities M3 and M4, the four‐cavity unit (M3, M4, T3, and T4 in orange) close its band gap to allow the wave motion from cavities M3 and M4 to cavities T3 and T4. Then, the wave signal will move along the orange cavity path on the top layer. The expected wave motion within this I‐pattern waveguide is verified by numerical simulations (12.52 kHz in Figure 4D and more frequencies in Figures S10A,B, Supporting Information). To further verify the waveguiding behavior, we carry out experiments by inputting acoustic signals in cavity M1 and measuring acoustic pressures in cavities P1‐P4 (Figure 4D–E). The experimental results show that the measured acoustic pressure in cavity P1 is much higher than those in cavities P2‐P4 within 12.45–12.7 Hz (gray shaded region in Figure 4E), indicating that the wave signal selectively transports to cavity P1 within the designated frequency range.

Next, taking the Y‐pattern waveguide as the second example, we expect the acoustic signal within the non‐topological band gap (12.27–12.65 kHz in Figure 2A) can move along the orange path in Figure 4F: starting from the I‐path on the bottom layer and then bifurcating into two branches. The concept is verified by numerical simulations (Figures 4G and S10C,D, Supporting Information) and experiments within the frequency of 12.53–12.65 kHz (Figure 4H). Within 12.53–12.65 kHz, the acoustic pressures in cavities P1 and P2 are similar, and much higher than those in cavities P3 and P4 (Figure 4H). Comparing the effective frequency ranges in Figures 4E and 4H, we find a common frequency range within 12.53–12.65 kHz (orange shaded region), where two topological waveguides (I and Y patterns) can be freely switched by tuning the applied magnetic fields, without rebuilding the structures (Movie S3, Supporting Information).

### 2.4 Magnetoactive Topological Field‐Effect Wave Regulators

**Figure 5:**
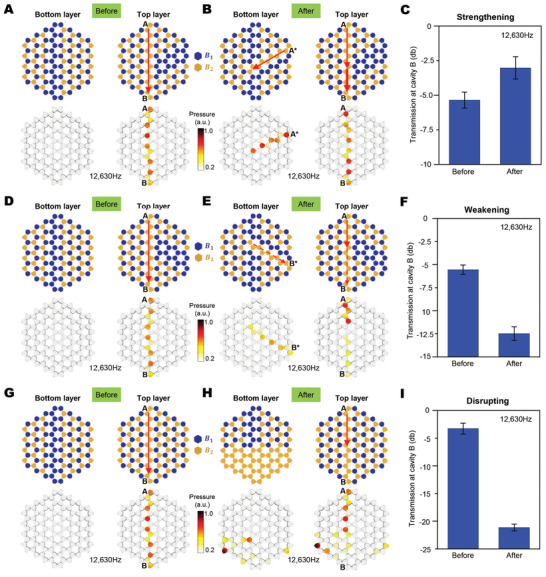
Magnetoactive topological field‐effect wave regulator. Harnessing the magneto‐actuated reconfiguration of air‐cavities in the bottom layer to regulate the topological wave transport on the top layer: A–C) strengthening, D–F) weakening, and G–I) disrupting. (A, D, and G) Schematics and numerical simulations of acoustic pressures of the top and bottom layers before regulating the bottom layer. (B, E, and H) Schematics and numerical simulations of acoustic pressures of the top and bottom layers after the respective regulations. (C, F, and I) Experimentally measured acoustic transmission at cavity B before and after the respective regulations. Experimental data represent the mean ± standard deviation of the mean with sample size *N* = 3–5 and *p*‐value < 0.01.

Besides waveguides, we also harness the proposed MATT to experimentally realize a class of topological field‐effect wave regulators (**Figure** **5**). The concept is to employ magnetic fields to reconfigure the cavities on the bottom layer to regulate the MATT on the top layer, but without changing the cavity configuration on the top layer. We here demonstrate reversible switching among three functions: strengthening (wave signal becoming stronger, Figures 5A–C), weakening (wave signal becoming weaker, Figures 5D–F), and disrupting (wave guiding being disrupted, Figures 5G–I). Before regulation, I‐pattern topological waveguides are prescribed on the top layer (Figure 5A,D,G): an acoustic wave moves starting from cavity A to cavity B on the top layer, without penetrating the bottom layer. In the example of strengthening, the cavity pattern on the bottom layer is reconfigured by controlling the applied magnetic fields, to install a half I‐pattern topological path (in orange color) starting from cavity A^*^ (Figure 5B). When two acoustic signals of the same amplitude and phase are input in both cavities A and A^*^, the acoustic signal from the bottom layer will move to the top layer and converge to cavity B, thus strengthening the acoustic pressure at cavity B (Figure 5B). This concept is verified by experiments that show a 31% pressure increase in cavity B after the regulation (12.63 kHz in Figure 5C). The weakening example follows a reverse idea: The cavity pattern on the bottom layer is reconfigured to install a half I‐pattern topological path (in orange color), such that the acoustic signal input in cavity A needs to bifurcate into two branches: to cavity B on the top layer and to cavity B^*^ on the bottom layer (Figure 5E). In this way, the acoustic pressure at cavity B is weakened after the regulation, which is verified by a 55% pressure drop in the experiment (12.63 kHz in Figure 5F). In the example of disrupting, a half fraction of cavities on the bottom layer are activated into the orange color to allow the acoustic wave to spread over on the bottom layer; thus, the selective topological transport to cavity B is disrupted (Figure 5H). The disrupting effect can be experimentally verified by a drastic pressure drop (88%) in cavity B (12.63 kHz, Figure 5I). Note that the numerical simulations and experiments are carried out for 12.63 kHz in Figure 5; more other frequencies within 12.4–12.65 kHz may also work, verified by the numerical simulations in Figure S11, Supporting Information. The above three regulation functions are achieved by simply controlling the magnetic fields in corresponding cavities on the bottom layer, but without rebuilding the structure or reconfiguring the top layer.

## 3 Conclusion

In summary, we present a class of MATTs capable of switching on and off acoustic topological states with magnetic fields. Integrating magneto‐tunable topological and non‐topological band gaps, in‐plane, and out‐of‐plane conductive routes can be on‐demand reconfigured without rebuilding the structures. As the first‐generation acoustic topological field‐effect transistor, the paradigm in this work may open the door of utilizing topological acoustics to analogize the broad functions of electronic field‐effect transistors, such as switching, stabilizing, amplifying, chopping, multiplexing, and current limiting.^[^ ^3^ ^]^ In addition, as field‐effect active non‐topological acoustic metamaterials have exhibited a promising potential to enable non‐local, rapid, and reversible modulation of acoustic properties,^[^ ^44^, ^45^, ^46^, ^47^, ^48^, ^49^, ^50^ ^]^ this work may promote the future research effort in integrating topological effects into field‐effect active acoustic metamaterials to realize new forms of topological field‐effect devices for on‐demand acoustic modulation, operation, and computation.

## 4 Experimental Section

### Fabrication of Acoustic Metamaterials

The acoustic metamaterials consisted of two components: housing and volume controllers. The housings were made of acrylic sheets (McMaster‐Carr, U.S.) layered on each other with specific patterns (Figures S1 and S8, Supporting Information). Plastic binding barrels (90249A640, McMaster‐Carr, USA) were used to assemble the acrylic sheets to form housings. There were 96 air cavities in the single‐layer structure (Figure S1, Supporting Information), and 192 in the double‐layer structure (Figure S8, Supporting Information). Each air cavity is in hexagonal‐prism shape with a lateral length of 14 mm and height of 19.05 mm. Two lateral cavities are connected via two cuboid air channels (cross‐section 3.35 mm × 3.18 mm, length 3.75 mm); two vertical cavities are connected via one cuboid air channel (cross‐section 3.45 mm × 3.55 mm, height 6.5 mm). A volume controller was bonded to the ground cover within each air cavity. Each switch was made of a hexagonal acrylic sheet (thickness 1.59 mm), the movable baffle, a magnet (D81AD‐P, K&J Magnetics), and an elastomer sponge. The elastomer sponge (thickness 2.5 mm) was fabricated by mixing and curing 22 g of silicon rubber (Mold Max 14NV, Smooth‐On), 60 g of fine sugar powder, and 10 g of ferromagnetic iron particles (Sigma Aldrich), followed by dissolving the sugar in water for 24 h. The acrylic sheet, magnet, and elastomer sponge were bonded with super glue (Gorilla Glue Company).

### Experimental Testing

Under each air cavity, a control magnet (D81AD‐P, K&J Magnetics) was employed to tune the effective volume of the air cavity. The applied magnetic field intensity was tuned by modulating the distance from the control magnet to the ground cover. The magnetic field was measured by a Gauss meter (GM‐1, AlphaLab Inc.). Using elastomer sponge is because of its two properties: spontaneous recovery of the deformation so that the induced deformation can be rapidly recovered once the applied magnetic field is reduced, and zero Poisson's ratio to ensure no lateral expansion during the compressive deformation.

The acoustic testing was carried out following the schematic shown in Figure S5, Supporting Information. An NI signal acquisition module (USB‐4431, National Instruments) and a Labview code were employed to control the signal processing. The acoustic signal was generated by a tweeter (1W‐8Ohm, UXCell) that was powered by a functional generator (PI‐8127, PASCO). The acoustic signal was received by microphones (378B02 with 426E01, PCB Piezotronics) connected to a signal conditioner (482C05, PCB Piezotronics). Because the diameter of the microphone (1/2 inch) was smaller than the size of the air cavity, the microphones were inserted in the air cavities to measure the signals during the experiments. The acoustic transmission in the unit of db was calculated as 10*log*_10_(*P*/*P*_0_), where *P* is the measured acoustic power and *P*_0_ is the incident acoustic power.

### Numerical Simulation

Numerical simulations were implemented with the acoustic module in COMSOL Multiphysics v5.3, a commercial finite element software. Since we assume that housings and switches are hard boundaries, only air cavities and their air channels are modeled. Dispersion relation analyses were implemented with unit cell models with Floquet periodic boundary conditions. For full‐model simulations, normal inward displacement was given as the input signal, and the displayed acoustic pressure amplitude was normalized (i.e., divided) by the maximum pressure amplitude at a specific frequency stated in each simulation case. All these simulations were validated by benchmark calculations, and the mesh accuracy was ascertained through a mesh refinement study.

### Statistical Analysis

The noise of the experimentally measured data was removed with Savitzky–Golay filter in MATLAB. The data in Figure 3G, S6C, S6F, S6I, S7C, and S7F, Supporting Information were presented as acoustic transmissions with the unit of db in functions of frequencies. The data in Figure 5C,F,I were presented as mean ± standard deviation of the mean of the acoustic transmissions. Means were compared by unpaired t‐test with Welch's correction to account for potentially unequal variances. P‐value was smaller than 0.01 and the sample size was 3–5. MATLAB was employed to carry out the statistical analysis.

## Conflict of Interest

The authors declare no conflict of interest.

## Supporting information

Supporting Information

Supplemental Movie 1

Supplemental Movie 2

Supplemental Movie 3

## Data Availability

The data that support the findings of this study are available from the corresponding author upon reasonable request.

## References

[1] L. J. Edgar , US Patent 1,745 1930, 175.

[2] J. Bardeen , W. H. Brattain , Phys. Rev. 1948, 74, 230.

[3] P. Valizadeh , Field Effect Transistors, A Comprehensive Overview: From Basic Concepts to Novel Technologies, Wiley, Hoboken, New Jersey 2016.

[4] X. Qian , J. Liu , L. Fu , J. Li , Science 2014, 346, 1344. 25504715 10.1126/science.1256815

[5] J. Liu , T. H. Hsieh , P. Wei , W. Duan , J. Moodera , L. Fu , Nat. Mater. 2014, 13, 178. 24362950 10.1038/nmat3828

[6] L. Fleet , Nat. Phys. 2015, 11, 5.

[7] W. G. Vandenberghe , M. V. Fischetti , Nat. Commun. 2017, 8, 14184. 28106059 10.1038/ncomms14184 PMC5263869

[8] J. L. Collins , A. Tadich , W. Wu , L. C. Gomes , J. N. Rodrigues , C. Liu , J. Hellerstedt , H. Ryu , S. Tang , S.‐K. Mo , Nature 2018, 564, 390. 30532002 10.1038/s41586-018-0788-5

[9] K. v. Klitzing , G. Dorda , M. Pepper , Phys. Rev. Lett. 1980, 45, 494.

[10] R. B. Laughlin , Phys. Rev. Lett. 1983, 50, 1395.

[11] C. L. Kane , E. J. Mele , Phys. Rev. Lett. 2005, 95, 226801. 16384250 10.1103/PhysRevLett.95.226801

[12] B. A. Bernevig , T. L. Hughes , S.‐C. Zhang , Science 2006, 314, 1757. 17170299 10.1126/science.1133734

[13] G. Ma , M. Xiao , C. T. Chan , Nat. Rev. Phys. 2019, 1, 281.

[14] X. Zhang , M. Xiao , Y. Cheng , M.‐H. Lu , J. Christensen , Commun. Phys. 2018, 1, 97.

[15] B. Xie , H.‐X. Wang , X. Zhang , P. Zhan , J.‐H. Jiang , M. Lu , Y. Chen , Nat. Rev. Phys. 2021.

[16] M. Z. Hasan , C. L. Kane , Rev. Mod. Phys. 2010, 82, 3045.

[17] D. Hsieh , D. Qian , L. Wray , Y. Xia , Y. S. Hor , R. J. Cava , M. Z. Hasan , Nature 2008, 452, 970. 18432240 10.1038/nature06843

[18] X.‐L. Qi , S.‐C. Zhang , Rev. Mod. Phys. 2011, 83, 1057.

[19] Z. Yang , F. Gao , X. Shi , X. Lin , Z. Gao , Y. Chong , B. Zhang , Phys. Rev. Lett. 2015, 114, 114301. 25839273 10.1103/PhysRevLett.114.114301

[20] A. B. Khanikaev , R. Fleury , S. H. Mousavi , A. Alu , Nat. Commun. 2015, 6, 8260. 26440700 10.1038/ncomms9260 PMC4600716

[21] Y.‐G. Peng , C.‐Z. Qin , D.‐G. Zhao , Y.‐X. Shen , X.‐Y. Xu , M. Bao , H. Jia , X.‐F. Zhu , Nat. Commun. 2016, 7, 2318. 10.1038/ncomms13368 PMC5114601 27834375

[22] R. Fleury , A. B. Khanikaev , A. Alu , Nat. Commun. 2016, 7, 11744. 27312175 10.1038/ncomms11744 PMC4915042

[23] Z. Zhang , Y. Tian , Y. Wang , S. Gao , Y. Cheng , X. Liu , J. Christensen , Adv. Mater. 2018, 30, 1803229. 10.1002/adma.201803229 30059167

[24] J. Lu , C. Qiu , L. Ye , X. Fan , M. Ke , F. Zhang , Z. Liu , Nat. Phys. 2017, 13, 369.

[25] J. Lu , C. Qiu , M. Ke , Z. Liu , Phys. Rev. Lett. 2016, 116, 093901. 26991176 10.1103/PhysRevLett.116.093901

[26] C. He , X. Ni , H. Ge , X.‐C. Sun , Y.‐B. Chen , M.‐H. Lu , X.‐P. Liu , Y.‐F. Chen , Nat. Phys. 2016, 12, 1124.

[27] C. He , H.‐S. Lai , B. He , S.‐Y. Yu , X. Xu , M.‐H. Lu , Y.‐F. Chen , Nat. Commun. 2020, 11, 2318. 32385317 10.1038/s41467-020-16131-w PMC7211004

[28] C. He , S.‐Y. Yu , H. Ge , H. Wang , Y. Tian , H. Zhang , X.‐C. Sun , Y. Chen , J. Zhou , M.‐H. Lu , Nat. Commun. 2018, 9, 4555. 30385775 10.1038/s41467-018-07030-2 PMC6212403

[29] J. Lu , C. Qiu , W. Deng , X. Huang , F. Li , F. Zhang , S. Chen , Z. Liu , Phys. Rev. Lett. 2018, 120, 116802. 29601733 10.1103/PhysRevLett.120.116802

[30] H. He , C. Qiu , L. Ye , X. Cai , X. Fan , M. Ke , F. Zhang , Z. Liu , Nature 2018, 560, 61. 30068954 10.1038/s41586-018-0367-9

[31] F. Li , X. Huang , J. Lu , J. Ma , Z. Liu , Nat. Phys. 2018, 14, 30.

[32] M. Xiao , W.‐J. Chen , W.‐Y. He , C. T. Chan , Nat. Phys. 2015, 11, 920.

[33] H. Xue , Y. Yang , F. Gao , Y. Chong , B. Zhang , Nat. Mater. 2019, 18, 108. 30598539 10.1038/s41563-018-0251-x

[34] Y. Qi , C. Qiu , M. Xiao , H. He , M. Ke , Z. Liu , Phys. Rev. Lett. 2020, 124, 206601. 32501055 10.1103/PhysRevLett.124.206601

[35] X. Ni , M. Li , M. Weiner , A. Alù , A. B. Khanikaev , Nat. Commun. 2020, 11, 2108. 32355274 10.1038/s41467-020-15705-y PMC7193630

[36] M. Weiner , X. Ni , M. Li , A. Alù , A. B. Khanikaev , Sci. Adv. 2020, 6, eaay4166. 32258398 10.1126/sciadv.aay4166 PMC7101231

[37] X. Ni , M. Weiner , A. Alù , A. B. Khanikaev , Nat. Mater. 2019, 18, 113. 30598540 10.1038/s41563-018-0252-9

[38] X. Ni , K. Chen , M. Weiner , D. J. Apigo , C. Prodan , A. Alù , E. Prodan , A. B. Khanikaev , Commun. Phys. 2019, 2, 55.

[39] X. Zhang , H.‐X. Wang , Z.‐K. Lin , Y. Tian , B. Xie , M.‐H. Lu , Y.‐F. Chen , J.‐H. Jiang , Nat. Phys. 2019, 15, 582.

[40] X. Zhang , Z.‐K. Lin , H.‐X. Wang , Z. Xiong , Y. Tian , M.‐H. Lu , Y.‐F. Chen , J.‐H. Jiang , Nat. Commun. 2020, 11, 65. 31900420 10.1038/s41467-019-13861-4 PMC6941978

[41] Z. Zhang , Y. Tian , Y. Cheng , Q. Wei , X. Liu , J. Christensen , Phys. Rev. Appl. 2018, 9, 034032.

[42] J. P. Xia , D. Jia , H. X. Sun , S. Q. Yuan , Y. Ge , Q. R. Si , X. J. Liu , Adv. Mater. 2018, 30, 1805002. 10.1002/adma.201805002 30294812

[43] Z. Tian , C. Shen , J. Li , E. Reit , H. Bachman , J. E. Socolar , S. A. Cummer , T. J. Huang , Nat. Commun. 2020, 11, 762. 32034148 10.1038/s41467-020-14553-0 PMC7005747

[44] B.‐I. Popa , D. Shinde , A. Konneker , S. A. Cummer , Phys. Rev. B 2015, 91, 220303.

[45] L. Airoldi , M. Ruzzene , New J. Phys. 2011, 13, 113010.

[46] K. Yu , N. X. Fang , G. Huang , Q. Wang , Adv. Mater. 2018, 30, 1706348. 10.1002/adma.201706348 29638017

[47] G. Ma , X. Fan , P. Sheng , M. Fink , Proc. Natl. Acad. Sci. USA 2018, 115, 6638. 29891704 10.1073/pnas.1801175115 PMC6042092

[48] G. Ma , P. Sheng , Sci. Adv. 2016, 2, e1501595. 26933692 10.1126/sciadv.1501595 PMC4771441

[49] B. Assouar , B. Liang , Y. Wu , Y. Li , J.‐C. Cheng , Y. Jing , Nat. Rev. Mater. 2018, 3, 460.

[50] S. A. Cummer , J. Christensen , A. Alù , Nat. Rev. Mater. 2016, 1, 16001.

[51] F. Li , P. Anzel , J. Yang , P. G. Kevrekidis , C. Daraio , Nat. Commun. 2014, 5, 5311. 25354587 10.1038/ncomms6311

[52] T. Zhang , Y. Cheng , J.‐z. Guo , J.‐y. Xu , X.‐j. Liu , Appl. Phys. Lett. 2015, 106, 113503.

[53] Y. Wang , J.‐p. Xia , H.‐x. Sun , S.‐q. Yuan , X.‐j. Liu , Sci. Rep. 2019, 9, 1. 31175315 10.1038/s41598-019-44769-0 PMC6555849

[54] K. H. Lee , K. Yu , A. Xin , Z. Feng , Q. Wang , Research 2020, 4825185. 32110778 10.34133/2020/4825185 PMC7025040

