# Supplementary material for: Magnetoactive Acoustic Topological Transistors
Source: Adv Sci (Weinh). 2022 Apr 25;9(18):2201204. doi: 10.1002/advs.202201204 (PMC9218775; doi:10.1002/advs.202201204)
Supplement: Supplementary file 1 — Supporting Information [file ADVS-9-2201204-s002.pdf]

ADVANCED SCIENCE
Open Access

Supporting Information

for *Adv. Sci.*, DOI 10.1002/advs.202201204

Magnetoactive Acoustic Topological Transistors

*Kyung Hoon Lee, Hasan Al Ba'ba'a, Kunhao Yu, Ketian Li, Yanchu Zhang, Haixu Du, Sami F. Masri and Qiming Wang**

Supplementary Information for

Magnetoactive Acoustic Topological Transistors

Kyung Hoon Lee[1], Hasan Al Ba'ba'a[1], Kunhao Yu[1], Ketian Li[1], Yanchu Zhang[1], Haixu Du[1], Sami F. Masri[1], Qiming Wang[1*]

[1]Sonny Astani Department of Civil and Environmental Engineering, University of Southern California, Los Angeles, California 90089, United States.

*Correspondence to: qimingw@usc.edu (Q.W.)

1. Supplementary figures

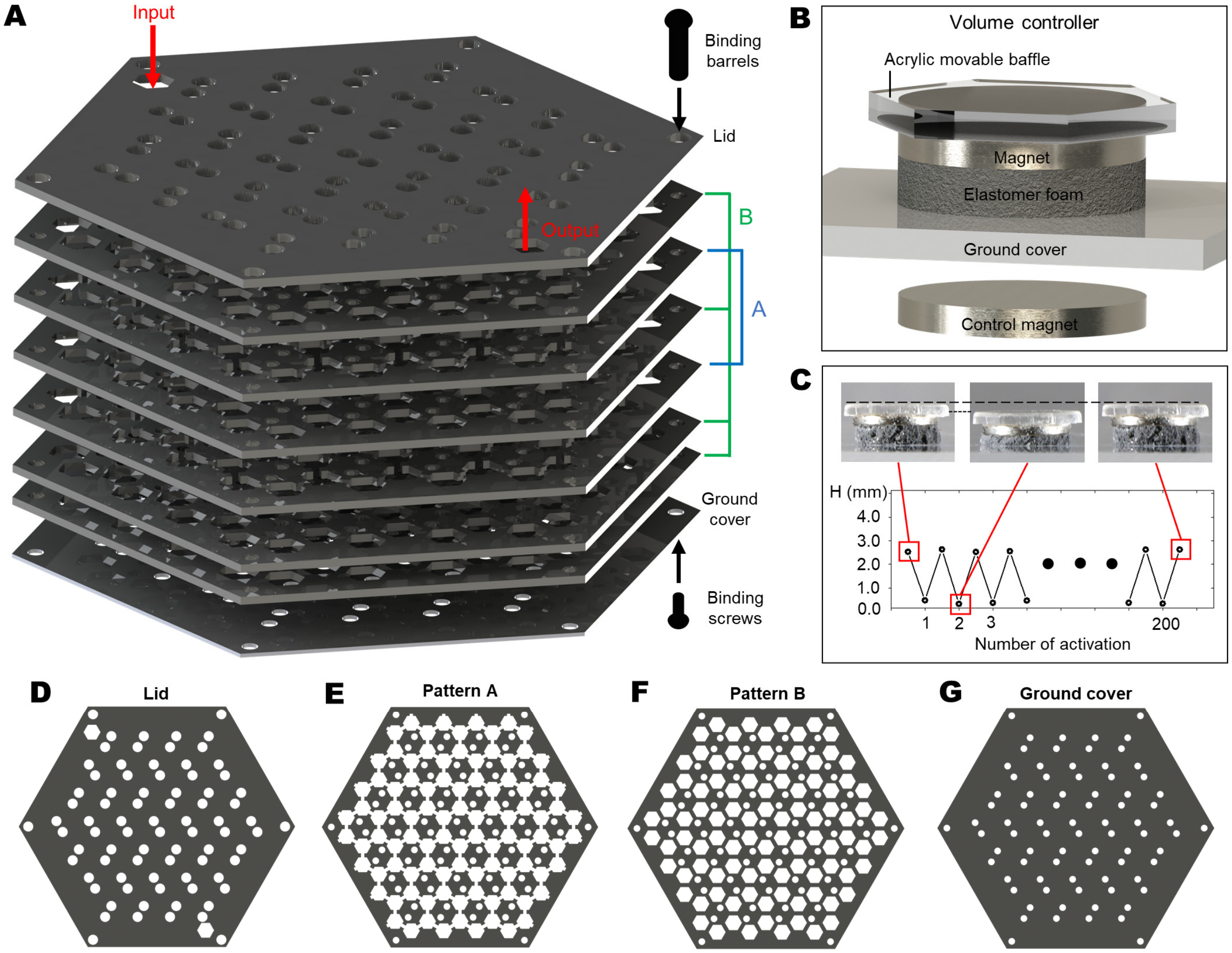

Figure S1. Schematics of single-layer air-cavity chamber structure. (**A**) An air-cavity chamber structure assembled with eight acrylic sheets and fastened by binding barrels and screws. (**B**) A volume controller made of an acrylic movable baffle, a magnet, and an elastomer foam. The control magnet is located outside of the air-cavity chamber to apply the magnetic field. When the control magnet is attached to the ground cover, the applied magnetic field is the highest, and the control magnet will not be easily detached by its weight due to the magnetic attraction. **(C)** The elastomer foam can recover its original shape even after more than 200 times of magnetic actuation. **(D-G)** Patterned acrylic sheets used to assemble the air-cavity chamber structure. The acrylic sheets are patterned and manufactured by a laser cutter.

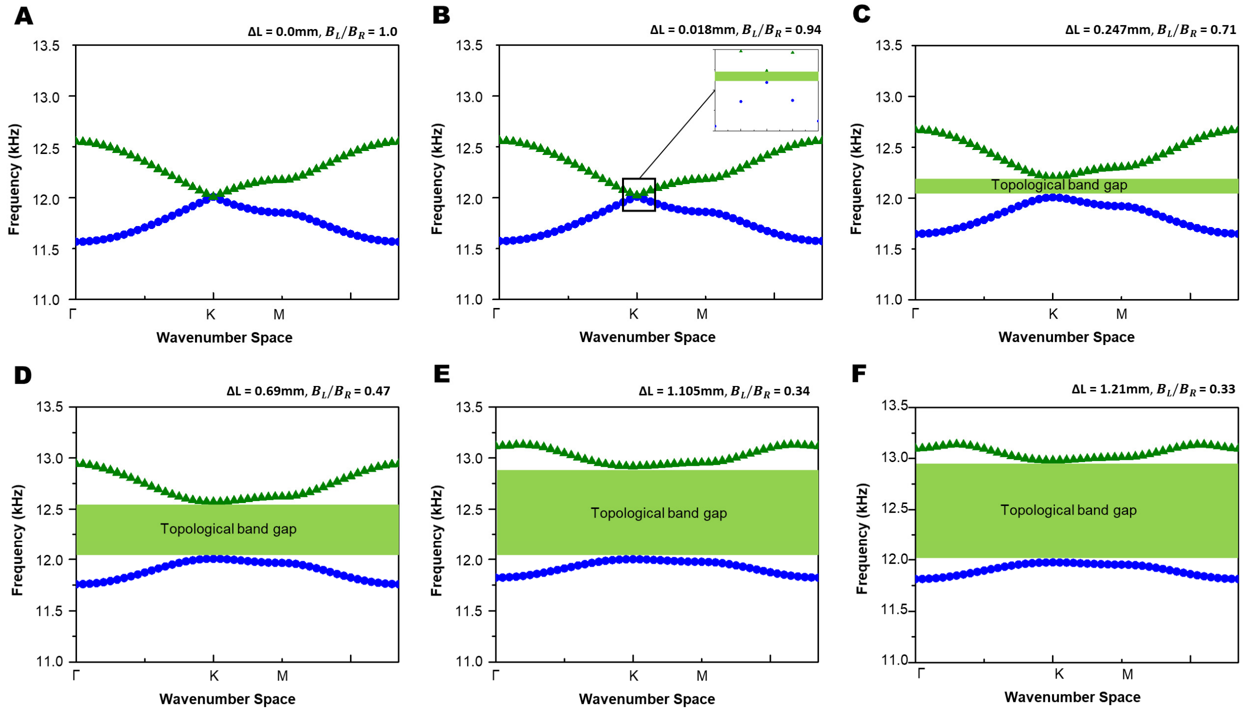

Figure S2. Numerically calculated dispersion relationships of the two-cavity unit cell with various magnetic field ratios: (**A**) $B_L/B_R = 1$, (**B**) $B_L/B_R = 0.94$, (**C**) $B_L/B_R = 0.71$, (**D**) $B_L/B_R = 0.47$, (**E**) $B_L/B_R = 0.34$, and (**F**) $B_L/B_R = 0.33$.

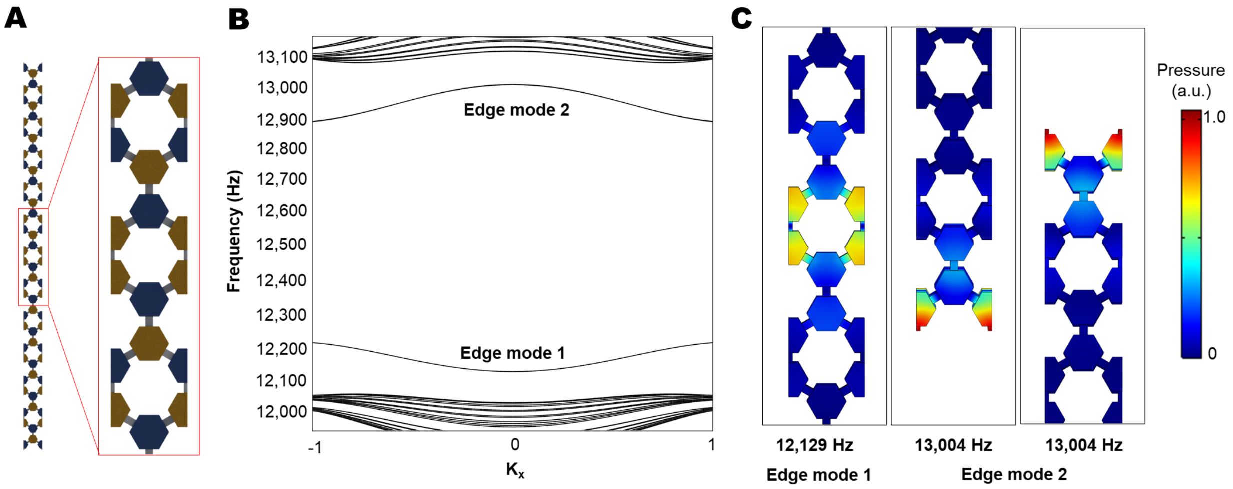

Figure S3. Supercell analysis to illustrate the edge modes. (**A**) A supercell model with a flipped pattern. (**B**) Dispersion relationship of the supercell. (**C**) Numerically simulated acoustic pressure amplitude to illustrate the edge modes. The displayed acoustic pressure amplitude was normalized by the maximum pressure amplitude at a specific frequency stated in each simulation case.

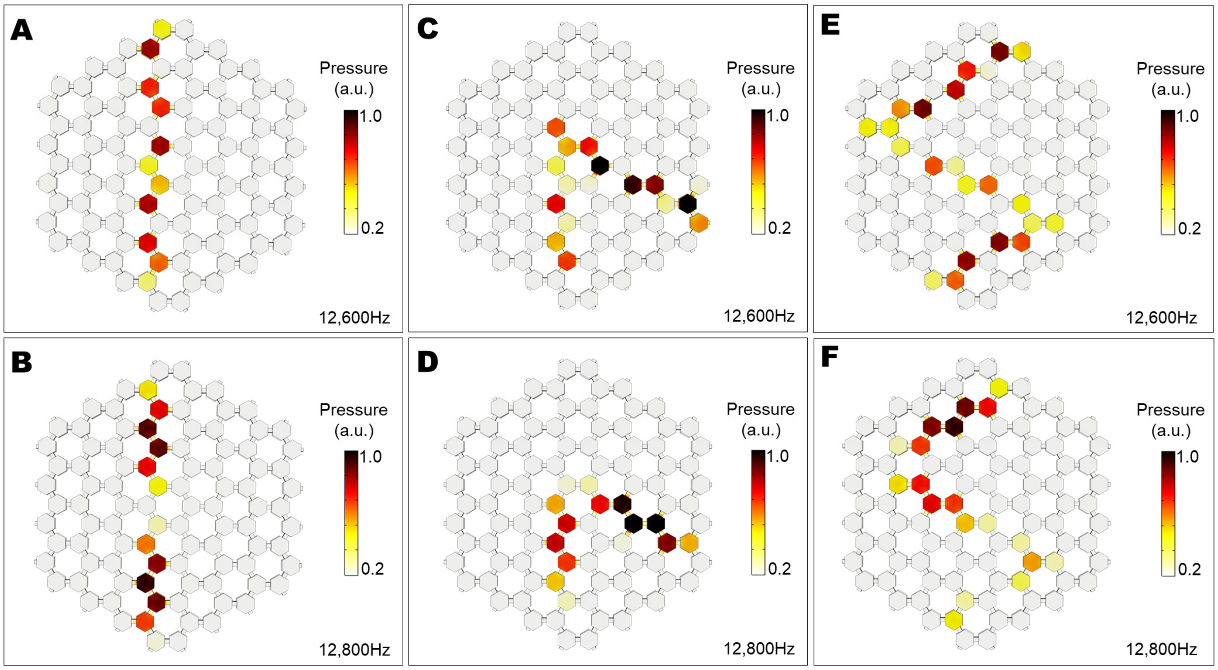

Figure S4. Numerical simulation of acoustic pressure within the single-layer air-cavity chamber at 12.6 kHz and 12.8 kHz. (**A**, **B**) I pattern waveguide, (**C**, **D**) V pattern waveguide, and (**E**, **F**) Z pattern waveguide.

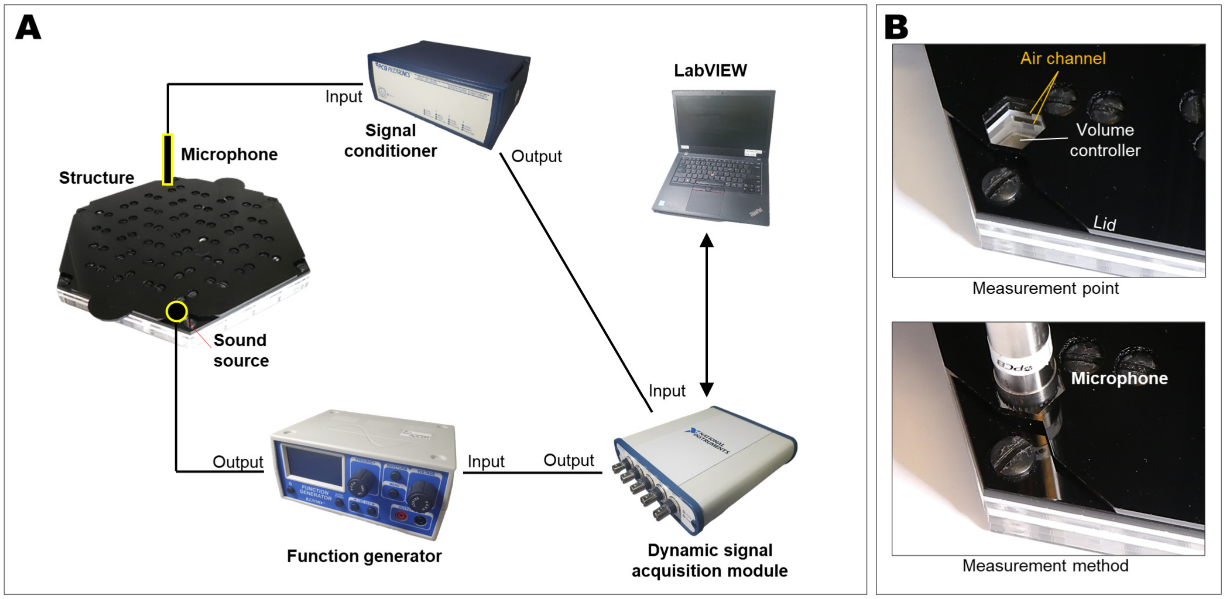

Figure S5. **Acoustic testing process**. **(A)** NI signal acquisition module (USB-4431, National Instruments) with LabVIEW code was employed to control the signal processing. The acoustic signal was generated by a tweeter (1W-8Ohm, UXCell) that was powered by a functional generator (PI-8127, PASCO). The acoustic signal was received by a microphone (378B02 with 426E01, PCB Piezotronics) connected to a signal conditioner (482C05, PCB Piezotronics). More than one tweeter and microphone are used depending on the type of tests. **(B)** When measuring acoustic signal, the microphone was inserted into the cavity slightly, not blocking the air channels. The dimension of the air cavity was designed to be larger than the size of the microphone.

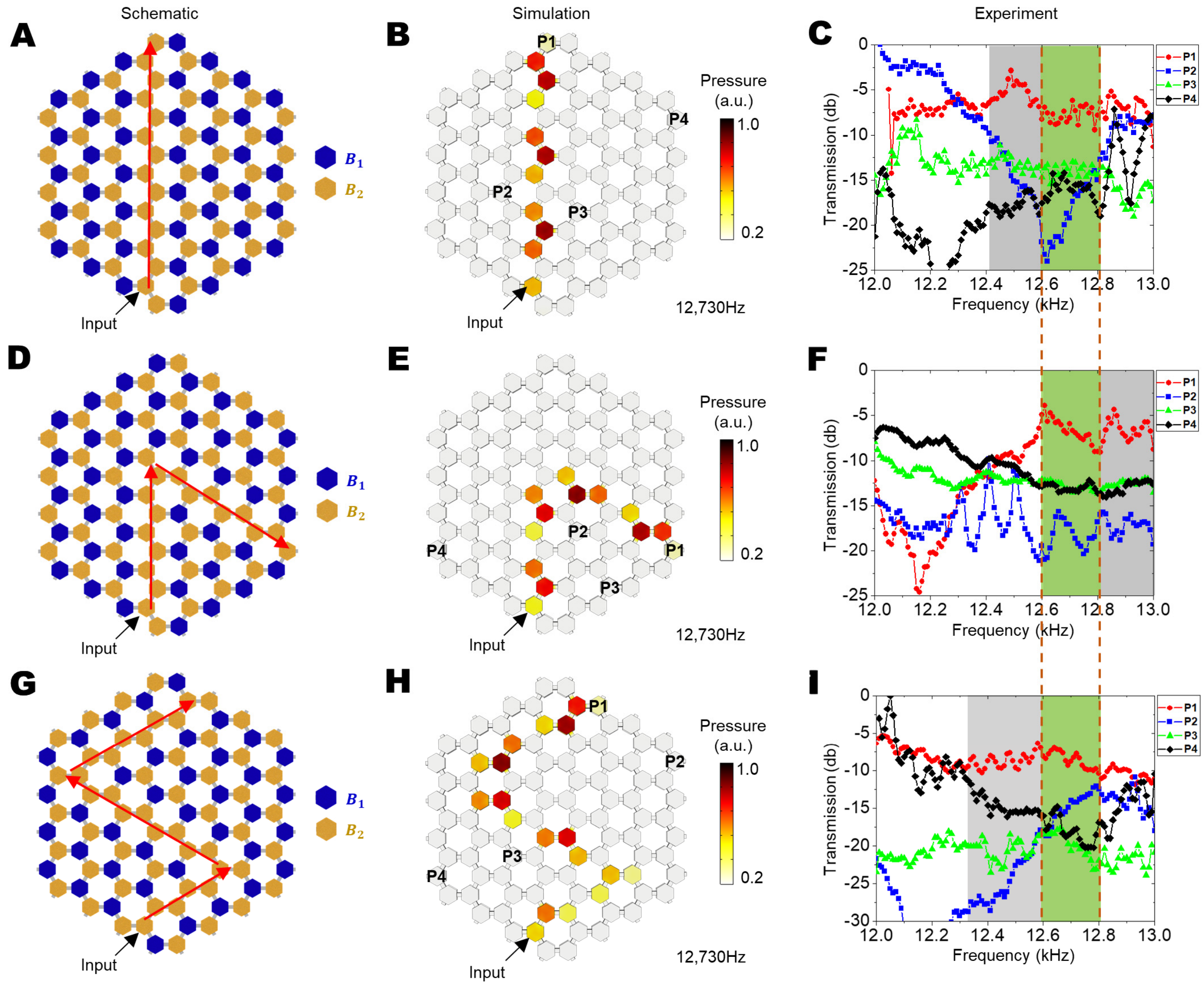

Figure S6. ATFET-enabled switching of in-plane conductive routes. Realization of (**A-C**) I pattern, (**D-F**) V pattern, and (**G-I**) Z pattern in-plane topological waveguides. (**A**, **D**, and **G**) Top-view schematics of air-cavity patterns. Blue and orange cavities are corresponding to the applied magnetic fields $B_1 = 0.087T$ and $B_2 = 0.265T$, respectively. (**B**, **E**, and **H**) Numerical simulations of the acoustic pressure within the air-cavity network. (**I**, **F**, and **I**) Experimentally measured acoustic transmission at cavities P1, P2, P3, and P4 in functions of frequencies. Gray shaded regions indicate the respective effective frequencies for each waveguide. Green shaded regions (12.6-12.82 kHz) indicate the frequency region effective for all three waveguides.

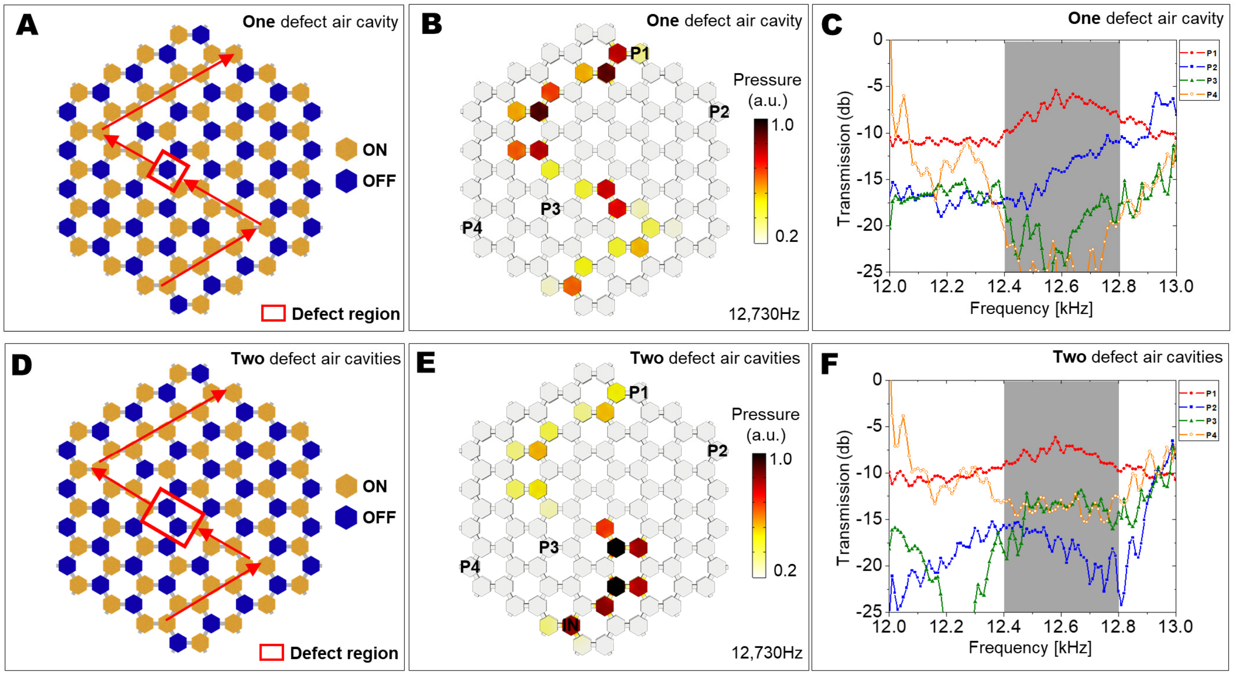

Figure S7. **Topological transport with high tolerance against structural defects**. (**A-C**) Tolerance against one deactivated air cavity: (**A**) air cavity layout, (**B**) numerical simulation of the acoustic pressure, and (**C**) experimentally measured acoustic transmission. (**D-F**) Tolerance against two deactivated air cavities: (**D**) air cavity layout, (**E**) numerical simulation of the acoustic pressure, and (**F**) experimentally measured acoustic transmission. The gray shaded regions in (**C**) and (**F**) indicate the effective frequency regions.

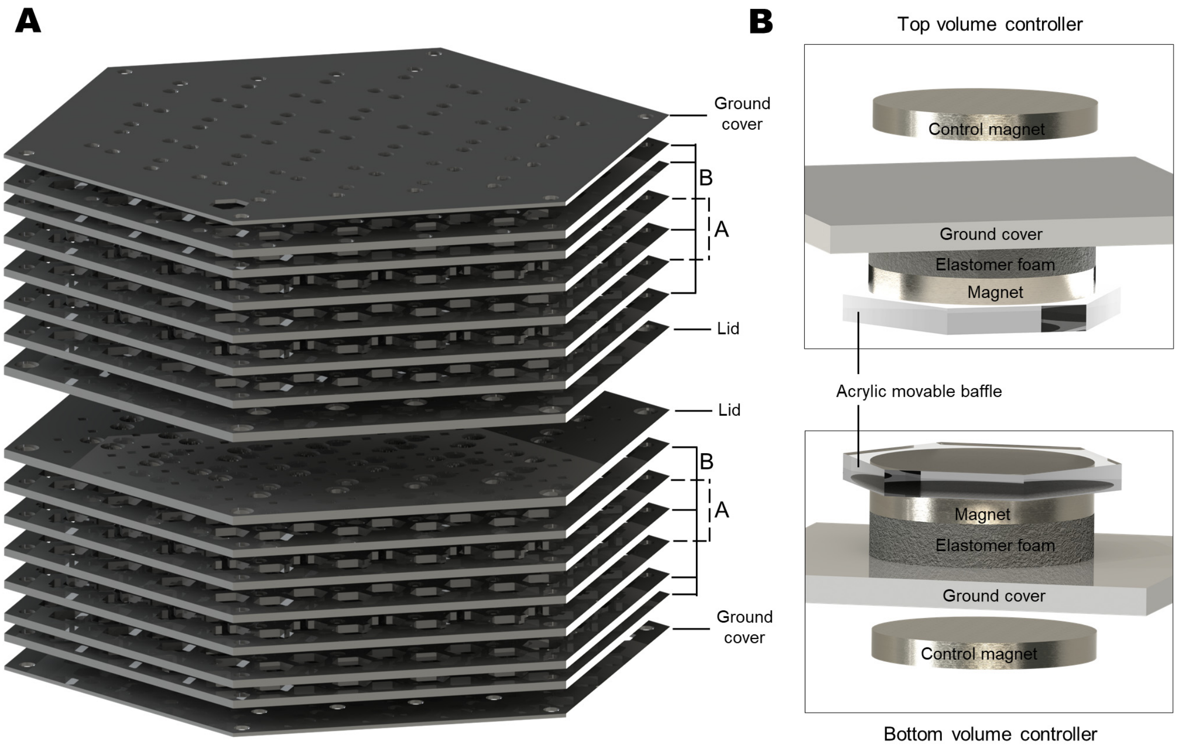

Figure S8. **Schematics of double-layer air-cavity chamber structure.** (**A**) Air-cavity chamber structure assembled with 14 acrylic sheets and fastened by binding barrels and screws. (**B**) Top and bottom volume controllers made of acrylic movable baffles, magnets, and elastomer foams.

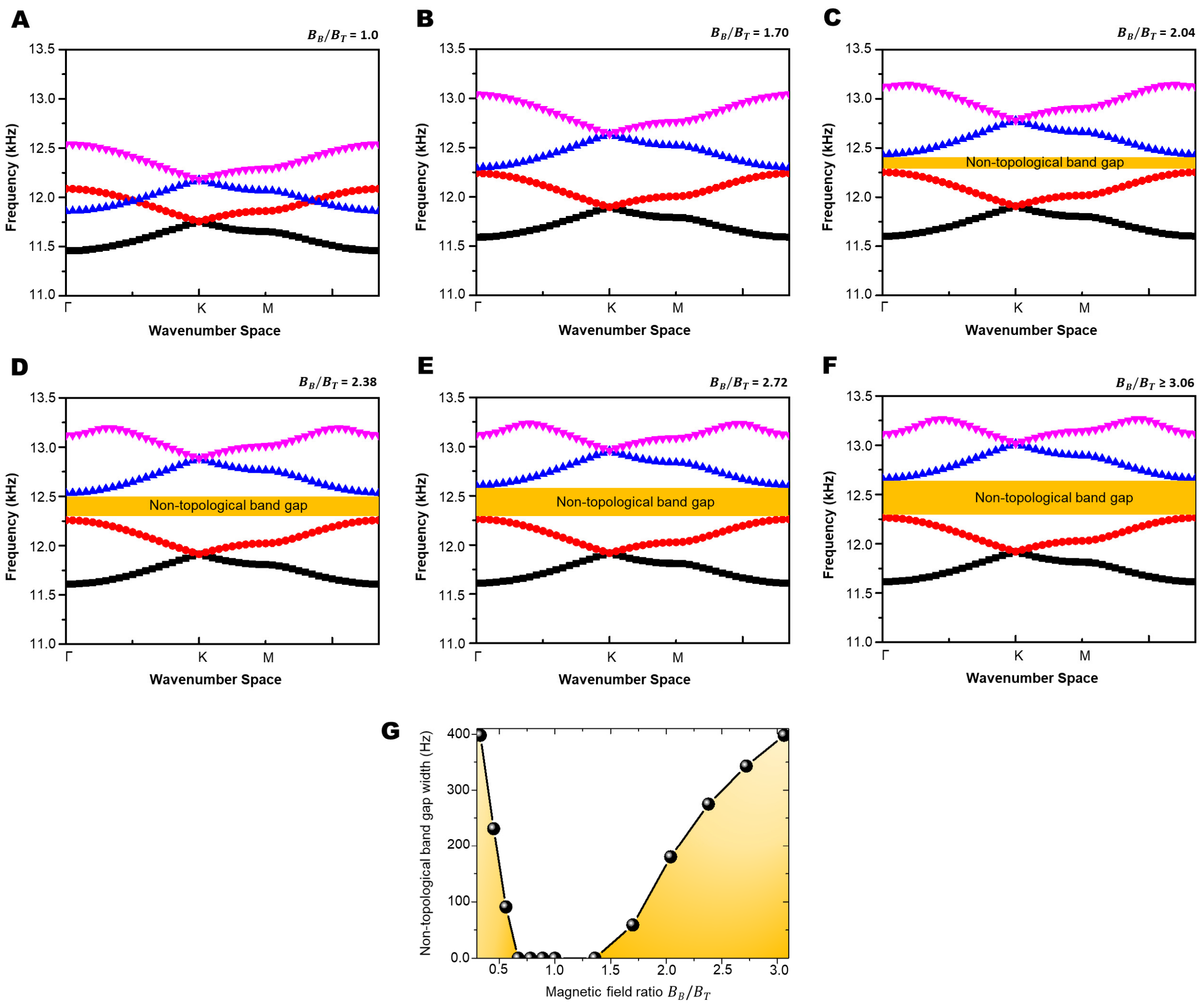

Figure S9. Numerically calculated dispersion relationships of the four-cavity unit cell with various magnetic field ratios. (**A**) $B_B/B_T = 1$, (**B**) $B_B/B_T = 1.70$, (**C**) $B_B/B_T = 2.04$, (**D**) $B_B/B_T = 2.38$, (**E**) $B_B/B_T = 2.72$, and (**F**) $B_B/B_T = 3.06$. (**G**) The numerically calculated non-topological band gap width in a function of the magnetic field ratio B_B/B_T.

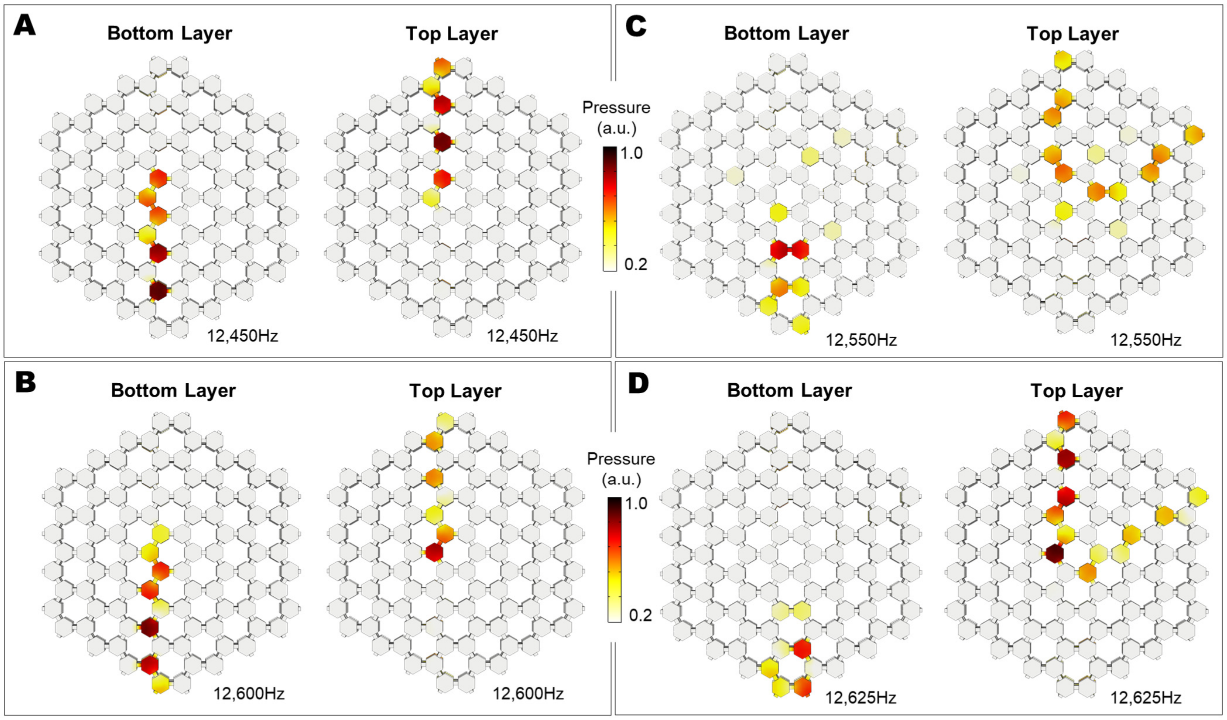

Figure S10. Numerical simulations of the acoustic pressure within the double-layer air-cavity chamber for (**A**, **B**) I pattern waveguide and (**C**, **D**) Y pattern waveguide at various acoustic frequencies. The cavity layouts of the I pattern waveguide and Y pattern waveguide are shown in **Figs. 4C** and **4F**, respectively.

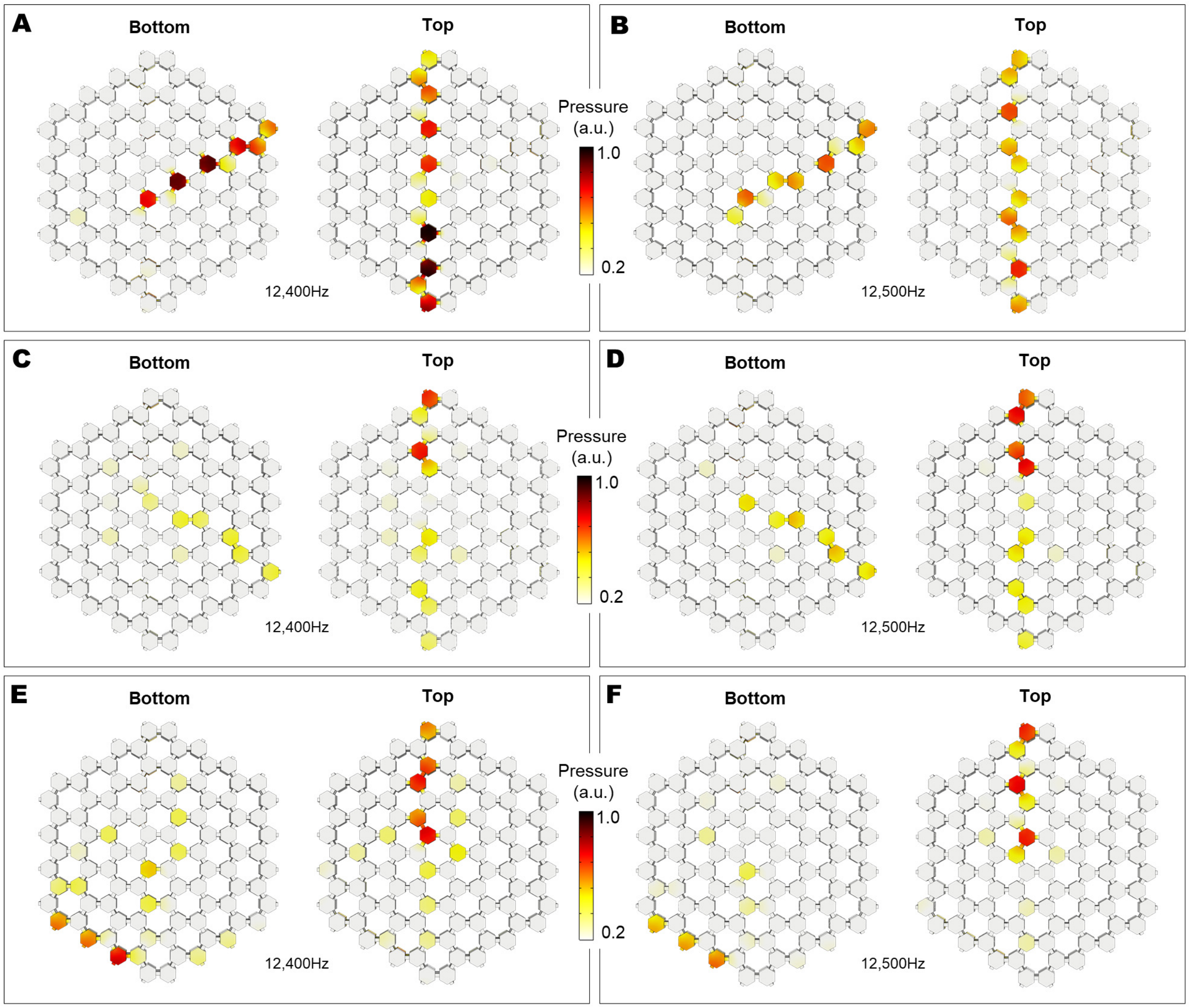

Figure S11. Numerical simulations of the acoustic pressure within the double-layer air-cavity chamber for (**A**, **B**) strengthening, (**C**, **D**) weakening, and (**E**, **F**) disrupting wave at different acoustic frequencies. The cavity layouts of three wave regulators are shown in **Figs. 5B, 5E**, and **5H**, respectively.

Supplementary movies

Movie S1: A movie to illustrate the fabrication and operation processes of the single-layer wave motion structure.

Movie S2: A movie to illustrate reversible switching among three topological waveguides (I, V, and Z patterns) in single-layer wave motion structure by reconfiguring the air-cavities with magnetic actuations.

Movie S3: A movie to illustrate reversible switching between two out-of-plane topological waveguides (I and Y patterns) in double-layer wave motion structure by reconfiguring the air-cavities with magnetic actuations.
